# Tidy up - The unfolded protein response in sepsis

**DOI:** 10.3389/fimmu.2022.980680

**Published:** 2022-10-20

**Authors:** Wolfgang Vivas, Sebastian Weis

**Affiliations:** ^1^ Department of Anesthesiology and Intensive Care Medicine, Jena University Hospital, Friedrich Schiller University, Jena, Germany; ^2^ Leibniz Institute for Natural Product Research and Infection Biology-Hans Knöll Institute (HKI), Jena, Germany; ^3^ Institute for Infectious Disease and Infection Control, Jena University Hospital, Friedrich Schiller University, Jena, Germany; ^4^ Center for Sepsis Control and Care, Jena University Hospital, Friedrich Schiller University, Jena, Germany

**Keywords:** sepsis, infection, unfolded protein response, immunity, endoplasmic reticulum stress, inflammation

## Abstract

Pathogens, their toxic byproducts, and the subsequent immune reaction exert different forms of stress and damage to the tissue of the infected host. This stress can trigger specific transcriptional and post-transcriptional programs that have evolved to limit the pathogenesis of infectious diseases by conferring tissue damage control. If these programs fail, infectious diseases can take a severe course including organ dysfunction and damage, a phenomenon that is known as sepsis and which is associated with high mortality. One of the key adaptive mechanisms to counter infection-associated stress is the unfolded protein response (UPR), aiming to reduce endoplasmic reticulum stress and restore protein homeostasis. This is mediated *via* a set of diverse and complementary mechanisms, *i.e.* the reduction of protein translation, increase of protein folding capacity, and increase of polyubiquitination of misfolded proteins and subsequent proteasomal degradation. However, UPR is not exclusively beneficial since its enhanced or prolonged activation might lead to detrimental effects such as cell death. Thus, fine-tuning and time-restricted regulation of the UPR should diminish disease severity of infectious disease and improve the outcome of sepsis while not bearing long-term consequences. In this review, we describe the current knowledge of the UPR, its role in infectious diseases, regulation mechanisms, and further clinical implications in sepsis.

## Introduction

Maintaining organ function while targeting invading pathogenic microorganisms is a balancing act for infected hosts (1). If successful, an infectious disease will likely take a milder course with no adverse effects on host tissues and the outcome will be favorable. If the measures taken by the host are maladaptive, organ dysfunction might occur and the infection will progress into sepsis, *i.e.* a diverse clinical syndrome that is still associated with a mortality rate of 30 to 50% despite available intensive care (2). Upon infection, hosts can mount two distinct but complementary defense mechanisms referred to as resistance to infection and disease tolerance to infection (3–5). Resistance mechanisms directly target pathogens aiming at reducing their number. Disease tolerance mechanisms aim at the preservation of tissue function and homeostasis. They rely on a set of tightly regulated stress and damage response pathways that sense and react to environmental cues, or infection-associated damage (6). If successful, tolerance pathways reduce disease severity without directly targeting pathogens (3–5). This also enforces restoration of homeostasis after pathogen clearance (6, 7) and directly counters sepsis-induced organ dysfunction.

The endoplasmic reticulum (ER) is a large and complex organelle with remarkable structural plasticity that serves as the main site for protein folding, maturation, and their subsequent transport to the Golgi apparatus in eukaryotic cells (8, 9). Thus, this organelle regulates essential cellular processes including calcium signaling, carbohydrate and lipid metabolism, and proteostasis (9, 10). Indeed, it carefully controls the rate of cellular protein synthesis and degradation under homeostatic conditions. However, even with the help of chaperones and folding enzymes, an accumulation of misfolded proteins in the lumen of the ER can occur, a process known as ER stress (10). Several conditions promote ER stress including calcium depletion, nutrient deprivation, hypoxia, inflammatory responses, or infection (10–13). Consequently, cells respond to ER stress by activating conserved adaptive signaling pathways–autophagy, ER-phagy, and the Unfolded Protein Response (UPR) (11–14). The UPR achieves proteostasis *via* i.) the reduction of protein translation, ii.) an increase of protein folding capacity, and iii.) an increase of polyubiquitination of misfolded protein and subsequent proteasomal degradation. This process is known as ER-associated protein degradation (ERAD) (15). Altogether, the UPR aims to counter the effects of proteotoxic stress and restore homeostasis. However, activation of the UPR is not only beneficial. Enhanced or prolonged activation of UPR can induce cell death and promote tissue damage (8, 16–18). Thus, disturbances in this delicate system have been shown to impact a wide range of pathological conditions, such as metabolic disease, cancer, inflammation, and infection (8, 19–21).

In the following chapters, we will discuss the molecular basis and regulation of the UPR and its role during inflammation and bacterial infection focusing on the immune system and several parenchymal organs. The findings from animal infection and inflammation models are summarized in **Table 1**.

**Table 1:** Summary of the UPR in animal studies with inflammatory stress (LPS) and/or infection.

Compartment	Model	Animal model	Outcome	Ref.
Lymphocytes	CLP	C57BL/6 WT	Apoptosis ↑ BiP ↑ CHOP ↑ XBP1 ↑	(22)
Spleen	CLP/LPS	B6.*Chop* ^-/-^ *vs.* WT	Survival ↑ Pathogen Load ↓ Caspase-3 activation ↓ Apoptosis ↓ IL10, TNF ↓	(23)
Small intestine	LPS	B6.*Pad4* ^-/-^ *vs.* WT	Intestinal injury ↓ NETs formation ↓ CHOP ↓ Inflammatory cytokine ↓ BiP and XBP1s ↓	(24)
Peritoneum	LPS-tolerance AND *P. aeruginosa vs.* LPS-tolerance AND *P.aeruginosa* + tunicamycin/thapsigargin	C57BL/6 WT	Inflammatory Cytokine ↑ GSK-3β activation ↑ Mortality ↓	(25)
Whole liver/Kupfer cells	Burn + LPS /CLP	Sprague-Dawley WT rats & C57BL/6 WT mice	Liver damage: serum ALT/AST ↑ Inflammasome activation ↑ ER stress ↑ BiP and CHOP ↑ Apoptosis ↑ Inflammatory cytokine↑ Altered hepatocytes transcriptional program	(26–29)
Lung	LPS/CLP	C57BL/6 WT	ER stress ↑ BiP, CHOP, p-eIF2α, ATF4, XBP1s ↑ Inflammatory cytokine ↑ Apoptosis ↑	(30, 31)
Kidney/renal tubular cells	LPS/CLP	C57BL/6 WT	XBP1 ↑ Serum creatinine/Blood urea nitrogen ↑ Kidney tubular necrosis ↑ CHOP ↑ Inflammatory cytokines ↑ PKR activation ↑ eIF2α-phosphorylation ↑ Protein translation ↓	(32, 33)
Heart	LPS/CLP	Sprague-Dawley WT rats	Cardiac injury ↑ Inflammatory cytokines ↑ BiP, GRP94, caspase-12, and CHOP ↑ BCL-2 ↓	(34, 35)
		B6.*Fundc1* ^-/-^ *vs.* WT	Troponin T, LDH, creatinine kinase ↑ Mitochondria viability, potential membrane ↓ ATF5, mtDNAj, ClpP, LonP1, CHOP, Hsp10, and Hsp60 ↑	
Skeletal muscle	LPS/CLP	Sprague-Dawley WT rats & Pigs	eIF4F-phosphorylation ↓ Protein translation ↓	(36, 37)

ALT, alanine transaminase; AST, aspartate transaminase; ATF, activating transcription factor; BCL-2, B-cell lymphoma 2; BiP, binding immunoglobulin protein; CHOP, C/EBP homologous protein; CLP, cecal ligation and puncture; eIF, eukaryotic initiation factor; ER, endoplasmic reticulum; GSK-3β, glycogen synthase kinase 3β; LPS, lipopolysaccharide; NET, neutrophil extracellular trap; PAD4, peptidylarginine deiminase 4; PKR, protein kinase R; TNF, tumor necrosis factor; XBP1, X-box binding protein 1; WT, wild type. ↓ : Decreased effect. ↑: Increased effect.

## Regulation of the unfolded protein response

**Figure 1:**
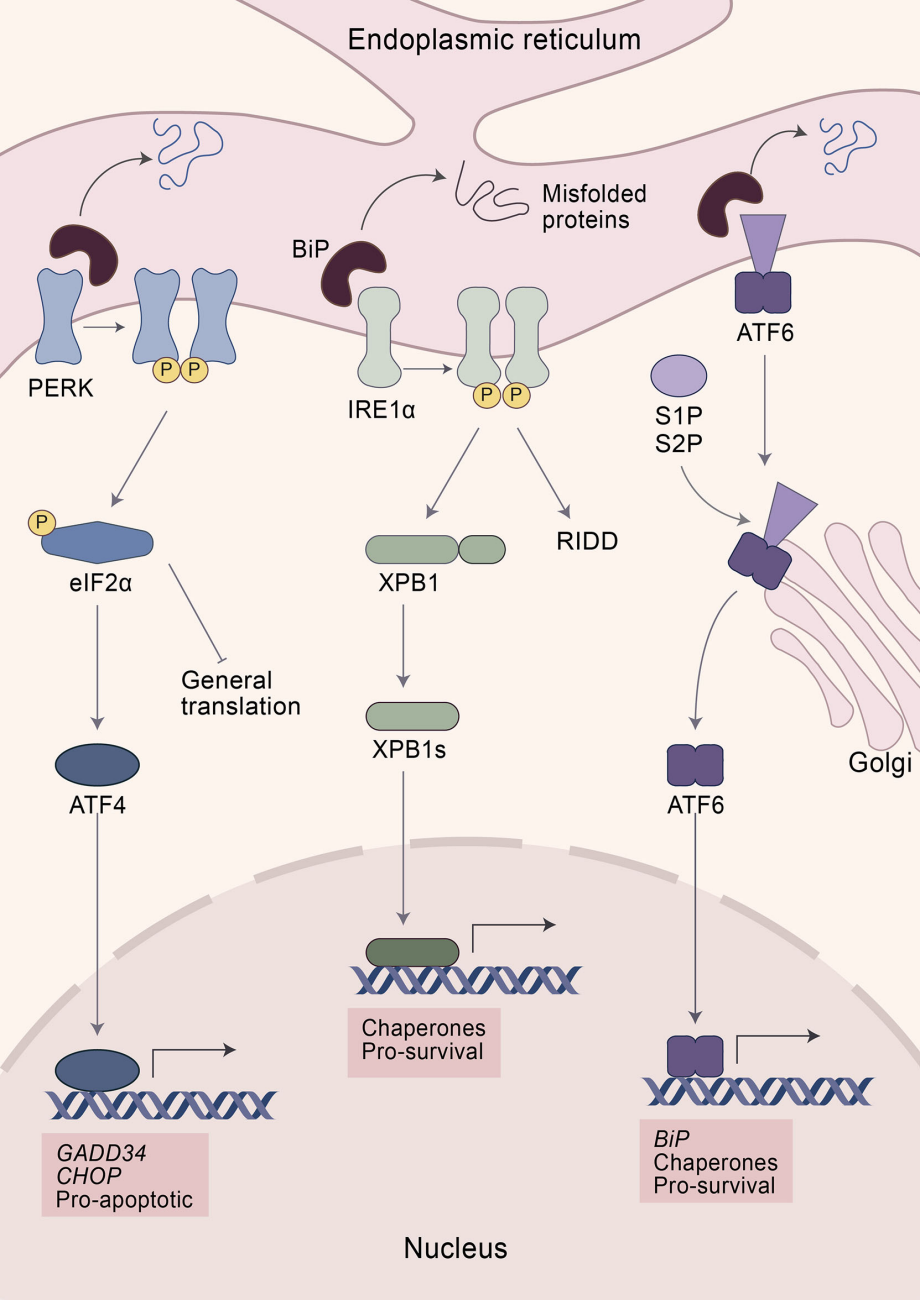
The unfolded protein response (UPR). Elevated levels of misfolded proteins are sensed by a group of specialized receptors in the ER—PERK, IRE1α, and ATF6—aiming at restoring proteostasis. The initial step in the recognition of misfolded proteins involves the dissociation of BiP from the UPR sensors resulting in their activation. PERK activation leads to the phosphorylation of eIF2α, which blocks the translation of 5´cap-mRNA while at the same time increasing the expression of ATF4. After restoring ER homeostasis, ATF4 promotes the expression of the transcription factor GADD34, which enhances the expression of the protein phosphatase 1 (PP1) which dephosphorylates eIF2α and restores protein translation. However, if ER stress persists, ATF4 induces apoptosis *via* CHOP. IRE1α activation reduces ER stress *via* two mechanisms: i.) degradation of mRNA by IRE1α-dependent decay and ii.) cleavage of XBP1 transforming it into its active form: XBP1s. XBP1s is a transcription factor that induces the expression of protein chaperones increasing the folding capacity of the ER. ATF6 activation promotes its translocation to the Golgi apparatus. There ATF6 is cleaved by two proteases—S1P and S2P—turning it into an active transcription factor. ATF6 enhances the expression of chaperones and *BiP*. While PERK is considered to be pro-apoptotic, IRE1α and ATF6 promote survival upon ER stress.

Until now, three main conserved molecular branches are identified that constitute the UPR. They operate in parallel *via* distinct signaling mechanisms and are named after their key-regulating proteins, i.e., i.) protein kinase R-like ER kinase (PERK); ii.) inositol-requiring enzyme 1-alpha (IRE1α); and iii.) activating transcription factor-6 (ATF6) (**Figure 1**) (8, 13, 38).

### The PERK branch

The initial step in the recognition of misfolded proteins involves the dissociation of Binding immunoglobulin Protein (BiP) from the UPR sensors resulting in their activation. Upon sensing ER stress, PERK oligomerizes within the ER and phosphorylates itself and its substrates, including the nuclear factor erythroid 2-related factor (NRF2) and the eukaryotic translation initiation factor 2-alpha (eIF2α) (13, 14, 39, 40). Phosphorylation of eIF2α halts protein translation *via* inhibition of the eIF2-GTP-Met-tRNA ternary complex (13, 39). However, eIF2α is also phosphorylated by other sensors including the double-stranded RNA-dependent protein kinase (PKR), heme-regulated eIF2α kinase (HRI), and general common derepressive 2 (GCN2) (41–45). Altogether, they form part of the Integrated Stress Response (ISR), an adaptive pathway that helps restore cellular homeostasis in response to diverse stresses, such as ER stress, heme deprivation, oxidative stress, heat shock, viral infection, glucose deprivation, and amino acid deprivation (41–43, 45–47). In consequence, *eIF2α* is a vital gene. Mice with a mutation at the eIF2α phosphorylation site died within a few hours after birth, underscoring the essential role in normal physiology and mammalian development (48–50). Despite halting translation, phosphorylation of eIF2α promotes the expression of certain transcription factors, including ATF4 (13, 48). ATF4 has an important role in regulating normal metabolic processes and acts as a master transcription factor during UPR. It has the capacity to form diverse homodimers and heterodimers, while also being regulated at the transcriptional, translational, and post-translational levels, which allows tailored responses toward different cellular stresses (47, 51). During stressful conditions, elevated translation of ATF4 facilitates the expression of stress-responsive genes, including the phosphatase growth arrest and DNA damage-inducible 34 (GADD34) and the transcription factor C/EBP homologous protein (CHOP) (13, 47). GADD34 is a co-factor that activates protein phosphatase 1 (PP1) which dephosphorylates eIF2α, acting as an important negative feedback loop to restore mRNA translation once the stress has been resolved (47, 52–54). However, persistent activation of the UPR leads to the expression of genes that control apoptosis such as *Chop*, encoding a transcription factor known to induce cell death *e.g.* upregulation of pro-apoptotic genes, enhancing expression of cell death receptor, or by destabilizing the homeostasis of the oxidative environment of the ER (13, 47, 55–58). CHOP, nevertheless, is not only induced by PERK, since its expression also depends on members of the other UPR branches, including ATF6 and XBP1, which highlights the intricate nature of the UPR and its different branches (59–64).

PERK can also phosphorylate ERF2, an essential transcription factor involved in cellular metabolic adaptation to oxidative stress (40, 65, 66). Upon phosphorylation, ERF2 dissociates from its repressor, the Kelch-like enoyl-COA hydratase (ECH)-associated protein 1 (KEAP1), which leads to NRF2 nuclear translocation and subsequently increases the expression of proteins with antioxidant activity (40, 65, 66). However, PERK also controls ERF2 expression by a mechanism that does not rely on direct phosphorylation. Indeed, activation of ATF4 is also necessary for sufficient expression and nuclear translocation of NRF2 in response to ER stress (67). Thus, PERK plays an essential role by coordinating adaptive signaling pathways involved in resistance against ER and oxidative stress.

### The ATF6 branch

ATF6 serves as the second branch of the UPR response. Upon activation, ATF6 translocates to the Golgi apparatus by vesicular transport (68, 69). At the Golgi apparatus it is cleaved by two proteases, membrane-bound transcription factor site-1 proteases (S1P) and S2P, resulting in an active transcription factor that regulates the expression of several genes including *Chop* and chaperones to alleviate protein misfolding (8, 61). Among the chaperones regulated by ATF6 is BiP, which plays an integral and critical role in the UPR by sensing misfolded proteins (13). Indeed, overexpression of *BiP* reduces the activation of UPR while its inactivation promotes ER stress (70, 71). BiP binds transiently to the luminal domain of the UPR receptors—PERK, ATF6, and IRE1α—and detaches again to bind nascent proteins in case unfolded proteins accumulate in the ER lumen. It is not completely understood whether misfolded proteins are sensed either by direct contact with the UPR receptors or indirectly through BiP dissociation (9, 70, 72–74).

### The IRE1α branch

IRE1α is a bifunctional enzyme that senses the accumulation of unfolded proteins, leading to its dimerization and autophosphorylation (9, 75, 76). Subsequently, IRE1α cleaves mRNA encoding the UPR-specific transcription factor, X-box binding protein 1 (XBP1) resulting in its active form spliced XBP1 (XBP1s) (9). XBP1s can increase the expression of chaperones and thereby enhances the protein folding capacity of the ER (8, 9). While it is mostly considered that XBP1s promotes cell viability, this molecule also contributes to cell death by controlling the expression of *Chop* (59, 60, 63, 64). Besides activation *via* IRE1α, XBP1 is also modulated *via* other mechanisms, including binding to forkhead box protein 01 (FOXO1) or phosphorylation by the mitogen-activated protein kinase (MAPK-14, also known as p38). Altogether, XBP1 has an impact on development, metabolism, and disease (77–79). For instance, overexpression of *XBP1* improves glucose metabolism in severely obese mice and in a mouse model of insulin deficiency or insulin resistance (79). Moreover, mice with hepatocyte-specific deletion of *Xbp1* develop insulin resistance and are prone to liver injury (77, 80). Similarly, XBP1 modulates lipid metabolism since selective deletion of *Xbp1* in the liver results in hypocholesterolemia and hypotriglyceridemia, together with modulation of lipogenic genes indicating that XBP1 is a regulator of lipogenesis (81).

However, IRE1α controls metabolism and apoptosis through the degradation of mRNAs in a process known as regulated IRE1-dependent decay (RIDD) (75, 82–87). For example, several genes involved in lipogenesis and lipoprotein metabolism, such as *Angptl3* and *Ces1*, are substrates of RIDD (87). Subsequently, suppression of RIDD reversed hypolipidemia in XBP1-deficient mice (87). In addition, IRE1α can degrade *via* RIDD several microRNAs that suppress the expression of CASP2, resulting in increased CASP2 protein levels (83, 85, 88). CASP2 is a pre-mitochondrial protease that cleaves the BH3-only protein BID resulting in activation of the BAX/BAX apoptosis pathway (83, 88). However, IRE1α overactivation also induces the expression of thioredoxin-interacting protein (TXNIP), which in turn activates the NLR familypyrin domain containing 3 (NLRP3) inflammasome, resulting in IL-1β secretion and apoptosis (85). Similarly, IRE1α can interact and phosphorylate the tumor necrosis factor receptor-associated factor 2 (TRAF2) and apoptosis signal-regulating kinase 1 (ASK1) (9, 89). This interaction results in apoptosis by activating the downstream targets from TRAF2 and ASK1, including the c-Jun NH2 terminal kinase (JNK) and p38 MAPK (9, 89).

## The unfolded protein response during inflammation and infection

Inflammation is the essential biological process that aims at controlling host homeostasis in response to infection. While we will here focus on inflammation in the context of infection, we would like to encourage readers to read a recent definition of inflammation that takes into account the broader aspect of inflammatory responses [reviewed in (90)].

Activation of inflammation requires the recognition of pathogen-associated molecular patterns (PAMPs) or endogenous signals such as damage-associated molecular patterns (DAMPs) by specialized molecules known as pattern-recognition receptors (PRRs) (91–93). Consequently, the mobilization of immune cells and soluble mediators—such as cytokines and chemokines—orchestrate the recognition, clearance, and resolution of the infection. This process results in the anciently defined characteristics of inflammation: redness, warmth, swelling, and pain. Once the infection is cleared, repair and restore mechanisms that promote return to homeostasis are activated, which include the expansion of immune cells with suppressive function and production of inflammation-resolving cytokines. These mechanisms are of paramount importance since prolonged hyperinflammatory responses can lead to host tissue damage (92). Thus, inflammation is a tightly controlled process that involves a network of cellular communication and intracellular signaling pathways. Understanding mechanisms that modulate inflammation in the context of infection is a major topic of research.

The UPR modulates inflammation by interacting with PRRs and their downstream inflammatory signaling pathways, including nuclear factor kappa-light-chain-enhancer of B cells (NF-κB), activator protein 1 (AP-1), and interferon regulatory factors (IRFs) *via* diverse mechanisms [reviewed in (94)]. These include: induction of specific IκB degradation and NF-κB nuclear translocation, transcriptional repression of negative regulators of NF-κB *via* CHOP, activation of AP-1 through MAPKs, or phosphorylation and activation of IRF3 (94).

Consequently, the UPR impacts the differentiation and function of several types of innate and adaptive immune cells. For example, Martinon et al. showed that activation of TLR2 and TLR4 in macrophages triggers IRE1α-XBP1 pathway activation but does not increase the mRNA expression of *Chop* or the chaperones *BiP* and *Erdj4*. Moreover, the IRE1α-XBP1 pathway is required for the optimal production of cytokines and chemokines, since using target-specific siRNA dampened *Il6*, *Tnf*, *Isg15*, *Ifnb*, *Il1b*, *and Rantes* mRNA expression (95). Also, activation of the nucleotide-binding oligomerization domain (NOD)-like receptor 2 (NOD2) signaling promotes the binding of laccase domain containing-1 (LACC1) to all three UPR receptors, resulting in increased cytokine production and a more efficient bacterial clearance by macrophages (96). In this line, Keestra et al. showed that macrophages derived from mice with constitutive deletion of *Nod1* and *Nod2* (*Nod1^-/-^Nod2^-/-^*) had lower expression and production of IL-6 in response to *Brucella abortus* infection or treatment with thapsigargin—an inhibitor of the Sarco/endoplasmic reticulum Ca^2^ ATPase, which results in calcium depletion and ER stress (97). Interestingly, the authors found that *Brucella abortus* induces ER stress *via* the release of the toxin VceC, a virulence factor that binds to BiP (98). Also, other toxins produced by bacteria, such as the *Escherichia coli*-derived Subtilase cytotoxin, can induce ER stress *via* cleavage of BiP, ultimately inducing apoptosis (99, 100). In a different study, infection of macrophages with the *Brucella abortus* strain RB51 promoted the production of reactive oxygen species (ROS) accompanied by the release of mitochondrial DNA and cytochrome c, resulting in mitochondrial dysfunction (101). Consequently, infected macrophages showed elevated activation of the (NLRP3) inflammasome and IL-1β release, which was blocked by inhibiting IRE1α with 4µ8C. Furthermore, the authors found that caspase 2 is responsible for ER stress-induced NLRP3 inflammasome activation, integrating cellular stress and innate immunity (101).

Similarly, the IRE1α-XBP1 pathway was shown to control the development and function of dendritic cells (DCs), professional antigen-presenting cells of the innate immune system that orchestrate the initiation of adaptive immunity. Already at steady state, CD8^+^ conventional DCs contain XBP1s (102). Further studies showed that loss of XBP1 leads to a reduction in DC frequency by increasing the levels of apoptosis (103). However, a study by Tavernier et al. showed that *Xbp1* deficiency can be compensated by overexpression of IRE1 enhancing RIDD in a cell type-specific manner (104). Exemplarily, RIDD activity counteracted Xbp1-induced apoptosis in intestinal DCs but not in lung DCs (104). Similarly, DCs levels were reconstituted when XBP1 was overexpressed in hematopoietic progenitors (103). Even further, upon infection with *Toxoplasma gondii*, XBP1 was required for IL-12 production and antigen presentation (102). These observations suggest an involvement of the IRE1α-XBP1 signaling pathway in the development and function of DCs.

Early work showed that the differentiation of B cells into plasma cells as well as antibody production also involves the activation of the UPR (105–107). Indeed, mice with a specific deletion of *Xbp1* in lymphocytes (*Xbp1-Rag2^-/-^*) had a deficient antibody secretion in response to LPS stimulation (107). This phenomenon was reversed when levels of spliced XBP1 were restored (108), experimentally proving the necessity of XBP1 for appropriate B cell function. How far this also involves other UPR factors needs to be further studied. Some data suggest that ATF6 could play a role since the application of LPS to CH12 B cell lymphoma promoted its cleavage, which can be interpreted as a surrogate marker for UPR activation (106, 109).

In summary, the UPR is involved in the host inflammatory response *via* crosstalk with several signaling pathways that modulate the differentiation and function of innate and adaptive immune cells.

## The unfolded protein response during sepsis

In the following section, we will discuss how the UPR contributes to bacterial sepsis in the context of the immune system and several parenchymal organs. Most of this knowledge is obtained from animal experimentation and summarized in **Table 1**.

### Immune system

The UPR is activated during the acute inflammatory response. Thus, it is not surprising that the UPR also affects the outcome of infectious processes, including sepsis. Indeed, a compiling body of evidence showed alterations in the UPR in septic patients. For example, gene expression of UPR genes correlated with the development of organ failure and endothelial dysfunction in septic patients (110).

A subset of patients that suffer from sepsis develops immunosuppression that accounts for the increased susceptibility to secondary infections (111). Some mechanisms for sepsis-induced immunosuppression include expansion of regulatory T cells, T cell exhaustion, impaired function of macrophages, and apoptosis in a diverse type of immune cells (111). Indeed, apoptosis is present in B cells and T cells in septic patients. B cells showed an exhausted-like/immunosuppressive phenotype characterized by low levels of MHC class II and elevated production of the suppressive cytokine IL-10 (112, 113). The UPR might play a role in these phenomena. This notion is supported by data from Ma et al. that showed that 24 hours after cecal ligation and puncture (CLP)—a surgery that induces polymicrobial peritonitis leading to sepsis—lymphocytes had elevated levels of apoptosis and expression of UPR genes, *e.g.*, *BiP*, *Xbp1s*, and *Chop* (22). However, expression of *CHOP* appears to have a negative effect on bacterial infection. Mice with a constitutive *Chop* knock-out (*Chop^-/-^)* had increased resistance to CLP (23). This was displayed by increased survival associated with a decreased host-pathogen load and lower plasma levels of tumor necrosis factor (TNF) and IL-10 when compared to wild-type (WT) mice after CLP (23).

Abnormal activation of immune cells during sepsis could lead to elevated UPR and tissue damage. For example, intestinal samples from patients with abdominal sepsis showed elevated levels of neutrophil extracellular traps (NETs) formation, enhanced apoptosis, and expression of *Chop and BiP*. To confirm these findings, a lethal dose of LPS was injected into wild-type and peptidylarginine deiminase 4 knock-out *(Pad4^-/-^)* mice, which cannot produce NETs. When compared to WT mice, *Pad4^-/-^* mice had better survival, reduced inflammation, lower tissue damage, and UPR gene expression. Accordingly, inhibition of the UPR by using 4-phenylbutyrate (4-PBA) alleviated NETs-induced damage to intestinal epithelial cells (24).

While the enhanced or prolonged activation of the UPR appears to be associated with a worse disease outcome during infection and sepsis, timed UPR activation can restore immune functions to reduce mortality against secondary infections. In a study by Kim et al., the authors injected mice with low doses of LPS to induce LPS tolerance, characterized by low production of inflammatory cytokines and increased susceptibility to secondary infection by *Pseudomonas aeruginosa.* However, treatment with ER stress agonists in the initial step of infection alleviated lung injury of septic mice subjected to *Pseudomonas aeruginosa* pneumonia *via* restoration of inflammatory cytokine release. This effect was accompanied by reduction of bacterial burden, in a glycogen synthase kinase 3β (GSK-3β) and IRE1α-XBP1 dependent manner (25).

In summary, these studies show that targeting the UPR in the context of sepsis could be an attractive approach to addressing altered activation and function of immune responses. However, care should be taken at the time of infection and in the context of secondary infections.

### Kidney

Sepsis-associated acute kidney injury (AKI) is the most common complication observed during sepsis and is directly associated with long-term morbidity and mortality, with little to no available specific treatment apart from organ replacement therapy (114, 115). Emerging studies have shown a promoting role of the UPR in the development of AKI during sepsis. Exemplarily, Ferré et al. showed that XBP1s was specifically elevated in kidneys of mice injected with LPS or subjected to CLP but not in different genetic models of chronic renal injury such as diabetes and polycystic kidney disease (32). Renal-tubular specific overexpression of *Xbp1s* enhanced expression of UPR genes such *as BiP* and *Chop.* Yet, in contrast to the expectation, a protective effect was not observed. Instead, this manipulation resulted in increased kidney injury as assessed by serum creatinine and blood urea nitrogen levels, tubular necrosis, and increased *Kim1* and *Ngal* expression. Upon LPS injection, kidney damage was even more pronounced. Consequently, mice with a renal-tubule specific deletion of *Xbp1 (Six2^Cre^Xbp1^-/-^*) were protected against LPS-induced kidney injury as evidenced by reduced expression of *Chop*, tissue dysfunction markers, and inflammatory molecules (32). In a time-course study, Hato et al. showed that protein synthesis in kidneys was elevated as early as 1 hour after LPS application, correlating to the acute inflammatory responses. In contrast, overall protein synthesis declined during the late phase of LPS response, which correlated to an increased level of protein kinase R (PKR), enhanced kidney damage, and distinct metabolic adaptations. However, by using an ISR inhibitor (ISRIB) in the early stages of sepsis, the authors were able to protect mice against the suppression of protein translation which resulted in decreased kidney injury (33).

In summary, these studies suggest that activation of UPR could be a driver of sepsis-associated AKI.

### Liver

The liver is a frequent target of dysfunctional inflammation (116). This organ hosts a range of cells, including endothelial, Kupffer, and hepatic stellate cells, that together play an essential role in a wide range of cellular processes, such as homeostasis, metabolism, and immunity (116, 117). During infection, however, these cells are primed and activated, resulting in the recruitment of immune cells to deal with the infection, which can lead to liver injury and progression to chronic liver failure (116, 118, 119). While not many studies have addressed the role of ER stress in liver injury during sepsis, rats subjected to septic burn had augmented inflammasome and UPR activation resulting in liver damage (26). In a different study, rats subjected to CLP showed elevated levels of apoptosis, enhanced markers for liver damage, altered morphological changes, and increased expression of UPR targets, including *Chop* (27). However, suppression of UPR activity *via* β-arrestin 1 or by using Berberine was sufficient to suppress the production of inflammatory cytokines, expression of UPR target genes, and liver damage (28, 120). Similarly, the UPR might play a role during hepatic ischemia-reperfusion injury, which is a common clinical complication from sepsis-associated liver dysfunction. In this regard, Rao et al. isolated Kupfer cells from mice with hepatic ischemia-reperfusion injury and observed an enhanced secretion of inflammatory cytokines, together with elevated expression of all three branches of the UPR when stimulated with LPS (121). Treatment with a siRNA against ATF6 was sufficient to protect liver tissue from damage. Finally, the regenerative capacity of the liver is crucial to support liver function during acute injury (122). Recently, the role of UPR during liver regeneration in the context of sepsis has been revealed (29, 123). Indeed, Dubois et al. showed that sepsis activates the expression of UPR target genes which blocks hepatic differentiation by modulating specific transcriptional programs. Consequently, inhibition of UPR activation was sufficient to restore hepatocyte regenerative capacity and reduce liver damage as evidenced by diminished serum aminotransferase levels (29).

In summary, these studies highlight that sepsis alters ER homeostasis and the resulting aberrant activation of UPR could be addressed to treat liver dysfunction during sepsis.

### Lung

Acute lung injury (ALI)/acute respiratory syndrome (ARDS) is frequently linked with sepsis (124, 125). This is associated with the loss of tissue integrity, and increased tissue permeability, surfactant dysfunction, and alveolar edema (124). It has now become clear that sepsis can induce lung injury in a direct or indirect way. Direct sepsis-induced ALI/ARDS arise from pulmonary infections, while indirect sepsis-induced ALI/ARDS arises from extrapulmonary infections (124, 125). Some studies indicate that altered UPR activation during sepsis underlies ALI/ARDS and might serve as a potential target to ameliorate these conditions. Indeed, this notion is supported by data showing that lung tissues from LPS-injected mice had an increased UPR activation and that reducing ER stress with 4-PBA alleviated NF-κB/HIF-1α activation (30). In concordance with this data, Chen et al. showed that septic mice had elevated ER stress associated with lung damage (31). However, preconditioning of the mice with the iron-containing DAMP heme (91, 126), that acts among others by activation of heme oxygenase (*Hmox/*HO)-1 expression—an essential enzyme in heme catabolism with potent anti-inflammatory properties (127)—protected animals by reducing UPR activation, and decreasing apoptosis in the lung (31). A potential role of heme/iron metabolism can be assumed since activation of HRI phosphorylates eIF2 resulting in an overall reduction of protein synthesis translation inhibition while activating ATF4-NRF2 to counter oxidative stress and apoptosis (128, 129). Of note, *Hmox-1* is a target gene of NRF2; thus, activation of the HRI-ATF4-NRF2-HO-1 axis could ameliorate pathogen-induced lung injury (129, 130).

ALI/ARDS is directly linked to the pathophysiological phenomenon of endothelial barrier dysfunction, which is frequently found in sepsis (131). Upon infection, the endothelium undergoes structural and functional changes. This adaptation, which is part of the adaptive host response, includes the release of cytokines, adhesion molecule expression, and altered permeability, which if dysfunctional can lead to the disruption of alveolar-capillary integrity and subsequent edema formation [reviewed in (132)]. Previous studies have shown that the UPR controls endothelial barrier function. Prolonged ER stress and subsequent UPR are associated with chronic vascular disease (133). However, in acute inflammatory stress and infection, mild ER stress was proposed to protect the endothelium by supporting endothelial function and consequently alleviating endothelial barrier dysfunction (134).

In summary, modulation of UPR activation has shown to be an interesting target to address sepsis-induced ALI/ARDS.

### Heart and skeletal muscle

Sepsis-induced myocardial dysfunction is a well-known feature of sepsis with a prevalence that varies from 10% to 70% (135). While it can induce profound contractile dysfunction, it is generally reversible (135). However, patients that recover from sepsis are at greater risk of recurrent heart failure (135–137). While several studies unveiled that septic-induced myocardial dysfunction involves impaired cardiovascular circulation, myocardial depression, impaired adrenergic pathways, and mitochondrial dysfunction (reviewed in (135, 137)), recent evidence suggests a critical involvement of the UPR. For example, Li et al. showed that rats subjected to CLP have elevated serum levels of creatine kinase and troponin, serological markers indicating muscle damage. Histological analyses revealed that the structure of septic hearts was altered while having elevated levels of apoptosis and expression of the UPR genes *BiP* and *Chop* (34). While these authors did not corroborate that inhibition of UPR activation can be beneficial against sepsis-induced myocardial injury, a different study by Zhang et al. showed that pre-conditioning of septic rats with cortistatin—a neuropeptide with immunosuppressive properties—reduced the expression of *Grp94*, *Chop*, and caspase 12, correlating with lower degrees of apoptosis (138).

**Figure 2:**
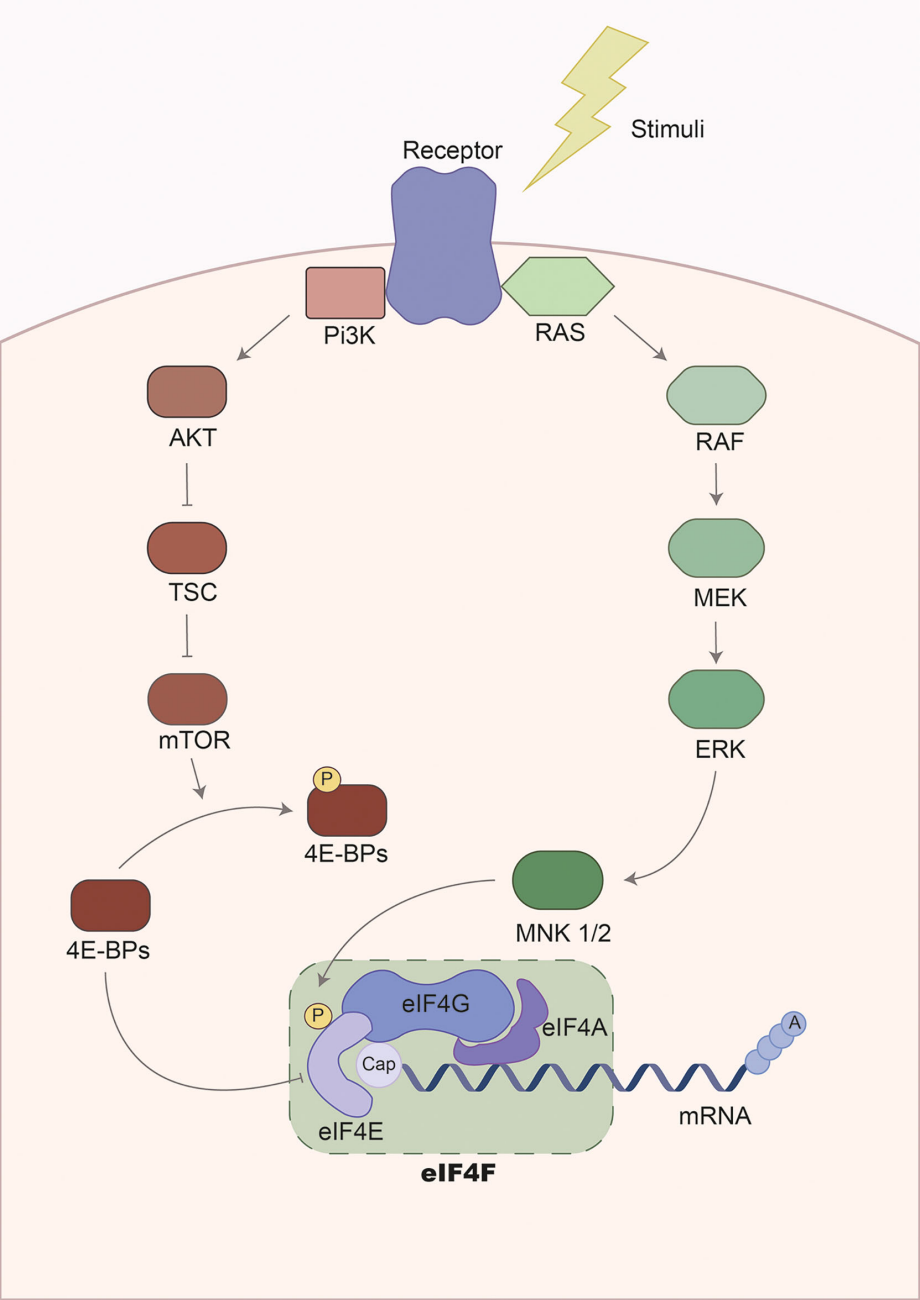
Regulation of the eukaryotic elongation factor 4F pathway. Gene expression is regulated during transcription and mRNA translation. The translation of the mRNA is mainly controlled in the initiation phase by the eukaryotic initiation factor 4F complex. This complex is composed of three subunits: i.) eIF4G, serving as the backbone and stabilizing other regulatory factors; ii.) eIF4A, a helicase involved in mRNA unwinding to facilitate recognition and translation by ribosomes; and iii.) eIF4E, recognizing and binding the 5´cap-mRNA. Consequently, the activity of this complex is modulated *via* phosphorylation by two major signaling cascades: the PI3K/mTOR and the MAPK/ERK pathways. On the one hand, the PI3K/mTOR signaling pathway regulates eIF4F *via* phosphorylation of eIF4E-binding proteins (4E-BPs). Initially, 4E-BPs binds to eIF4E suppressing translation initiation by disrupting the eIF4F complex. However, phosphorylation of 4E-BPs by the PI3K/mTOR promotes the release of 4E-BPs and allows translation initiation. On the other hand, activation of MAPK/ERK signaling leads to the phosphorylation of MNK1/2, a kinase that phosphorylates eIF4E. This results in translation initiation. The activation of signaling cascades is mediated by receptors activated by diverse extracellular stimuli, including cytokines, hormones, and growth factors.

Long-term debilitating features, such as muscle weakness are common after sepsis (139). The UPR and reduction of protein synthesis have also been linked to sepsis-induced muscle weakness (36, 140–143). Indeed, protein synthesis is impaired during sepsis partly by altering the initiation phase of mRNA translation *via* modulation of the eIF4F complex, i.e. the key regulator of the mRNA-ribosome recruitment phase of translation initiation (**Figure 2**). This complex is composed of several proteins, including i.) eIF4E, which binds to the mRNA 5´cap; ii.) eIF4A, a helicase that unwinds secondary structures facilitating the mRNA-ribosome interaction; and iii.) eIF4G, the backbone of the complex (144, 145). The activity of eIF4F is regulated by phosphorylation *via* diverse signaling cascades including PI3k/AKT/mTOR/4E-BPs and the RAS/RAF/MEK/ERK/MNK MAPK signaling pathways (145). In septic animals, however, eIF4F activity is impaired in skeletal muscle. Vary et al. showed that rats subjected to polymicrobial peritonitis by fecal slurry had diminished protein synthesis, together with reduced levels of eIF4G phosphorylation (36). The authors could restore eIF4G phosphorylation by inhibition of TNF and IL-1 receptors, suggesting that hyperinflammation-associated to sepsis leads to reduce protein synthesis *via* modulation of eIF4F activity (36). Moreover, rats subjected to CLP or pigs subjected to LPS had reduced levels of mTOR, 4E-BP1, and eIF4G phosphorylation, altering eIF4F complex formation and protein synthesis (37, 146).

Regarding the UPR, it was shown that patients with peritoneal sepsis had a higher expression of *XBP1*, but not of *ATF4* or *ATF6* in the muscle (142). This was associated with increased mRNA levels of the chemokine *CCL2* and the receptor *CD68*—a marker for macrophage infiltration in response to skeletal injury. In line with these findings, mice subjected to CLP also showed a higher expression of *Xbp1* in muscle tissues, but in contrast to humans, also of *Atf4*, and *BiP* (142). Of note, other animal studies had shown that ER stress and UPR counteract cancer-associated muscle wasting (147). Therefore, to date, it remains unclear whether increased UPR is an adaptive mechanism to counteract sepsis-induced muscle loss or whether it contributes to it differently than in the field of oncology.

## Future perspective

When appropriately activated, the UPR is a strong pillar of the adaptive stress response that decreases tissue dysfunction and damage during different types of diseases. However, exacerbated UPR activation during severe infections are in the majority of cases detrimental to the host. Currently, there are no drugs that specifically target the UPR to treat infectious diseases. A further and in-depth understanding of the regulation of the UPR in the context of severe infections including those leading to sepsis could help unveil novel host-directed therapeutic targets. Despite many years of research on the molecular level, there are still many open questions that need to be addressed in the future. Some of these are: What is the role of the UPR in the majority of clinically relevant infections caused by bacterial pathogens such as *Escherichia coli*, *Staphylococcus aureus*, and *Pneumococci* or viruses such as influenza or SARS-CoV-2? How and when does UPR activation lead to pathogenesis in the context of infection and could a controlled manipulation of this pathway be used to promote homeostasis during septic organ failure? How is the UPR regulated in human parenchymal tissues during the course of infectious disease and are there certain specific geno- or phenotypes that depend more on a functional UPR than others? Moreover, determining a precise timeline for the activation of UPR during sepsis might unveil unexpected results. If this is achieved, then pharmacological manipulation of the UPR likely in a time-dependent manner is an interesting approach to expand our treatment options for severe infectious disease in the clinical context. As such, we do think, that studying the UPR in infectious diseases is more than worthwhile.

## Author contributions

All authors listed have made a substantial, direct, and intellectual contribution to the work and approved it for publication.

## Funding

SW is currently funded by the Deutsche Forschungsgemeinschaft, DFG, project number WE 4971/6-1, the Excellence Cluster Balance of the Microverse (EXC 2051; 390713860), the Federal Ministry of Education and Research (BMBF) project number 01EN2001, and the Horizon 2020 FrameWork program grant 847422.

## Acknowledgments

We thank Dolly Montaño for her assistance in the preparation of figures and Dr. Dania Martínez, Dr. Gianna Hirth, Dr. Elisa Jentho, and Angelina Yershova for critical revisions of the manuscript.

## Conflict of interest

The authors declare that the research was conducted in the absence of any commercial or financial relationships that could be construed as a potential conflict of interest.

## Publisher’s note

All claims expressed in this article are solely those of the authors and do not necessarily represent those of their affiliated organizations, or those of the publisher, the editors and the reviewers. Any product that may be evaluated in this article, or claim that may be made by its manufacturer, is not guaranteed or endorsed by the publisher.

## Abbreviations

ATF, activating transcription factor; BiP, binding immunoglobulin protein; CHOP,C/EBP homologous protein; ER, endoplasmic reticulum; eIF2α,eukaryotic translation initiation factor 2-alpha; GADD34, DNA damage-inducible 34; IRE1α, inositol-requiring enzyme 1-alpha; PERK, protein kinase R-like ER kinase; UPR, unfolded protein response; XBP1, X-box binding protein 1.

## References

[1] Martins R Carlos AR Braza F Thompson JA Bastos-Amador P Ramos S. Disease tolerance as an inherent component of immunity. Annu Rev Immunol (2019) 37:405–37. doi: 10.1146/annurev-immunol-042718-041739 30673535

[2] Bauer M Coldewey SM Leitner M Loffler B Weis S Wetzker R. Deterioration of organ function as a hallmark in sepsis: The cellular perspective. Front Immunol (2018) 9:1460. doi: 10.3389/fimmu.2018.01460 29997622 PMC6028602

[3] Medzhitov R Schneider DS Soares MP. Disease tolerance as a defense strategy. Science (2012) 335(6071):936–41. doi: 10.1126/science.1214935 PMC3564547 22363001

[4] Råberg L Graham AL Read AF. Decomposing health: Tolerance and resistance to parasites in animals. Philos Trans R Soc Lond B Biol Sci (2009) 364(1513):37–49. doi: 10.1098/rstb.2008.0184 18926971 PMC2666700

[5] Schneider DS Ayres JS. Two ways to survive infection: What resistance and tolerance can teach us about treating infectious diseases. Nat Rev Immunol (2008) 8(11):889–95. doi: 10.1038/nri2432 PMC4368196 18927577

[6] Soares MP Gozzelino R Weis S. Tissue damage control in disease tolerance. Trends Immunol (2014) 35(10):483–94. doi: 10.1016/j.it.2014.08.001 25182198

[7] Kotas ME Medzhitov R. Homeostasis, inflammation, and disease susceptibility. Cell (2015) 160(5):816–27. doi: 10.1016/j.cell.2015.02.010 PMC4369762 25723161

[8] Lemmer IL Willemsen N Hilal N Bartelt A. A guide to understanding endoplasmic reticulum stress in metabolic disorders. Mol Metab (2021) 47:101169. doi: 10.1016/j.molmet.2021.101169 33484951 PMC7887651

[9] Adams CJ Kopp MC Larburu N Nowak PR Ali MMU. Structure and molecular mechanism of er stress signaling by the unfolded protein response signal activator Ire1. Front Mol Biosci (2019) 6:11. doi: 10.3389/fmolb.2019.00011 30931312 PMC6423427

[10] Schwarz DS Blower MD. The endoplasmic reticulum: Structure, function and response to cellular signaling. Cell Mol Life Sci (2016) 73(1):79–94. doi: 10.1007/s00018-015-2052-6 26433683 PMC4700099

[11] Bartelt A Widenmaier SB. Proteostasis in thermogenesis and obesity. Biol Chem (2020) 401(9):1019–30. doi: 10.1515/hsz-2019-0427 32061163

[12] Metcalf MG Higuchi-Sanabria R Garcia G Tsui CK Dillin A. Beyond the cell factory: Homeostatic regulation of and by the Upr(Er). Sci Adv (2020) 6(29):eabb9614. doi: 10.1126/sciadv.abb9614 32832649 PMC7439504

[13] Walter P Ron D. The unfolded protein response: From stress pathway to homeostatic regulation. Science (2011) 334(6059):1081–6. doi: 10.1126/science.1209038 22116877

[14] Yang M Luo S Wang X Li C Yang J Zhu X. Er-phagy: A new regulator of er homeostasis. Front Cell Dev Biol (2021) 9:684526. doi: 10.3389/fcell.2021.684526 34307364 PMC8299523

[15] Wu X Siggel M Ovchinnikov S Mi W Svetlov V Nudler E. Structural basis of er-associated protein degradation mediated by the Hrd1 ubiquitin ligase complex. Science (2020) 368(6489):1–32. doi: 10.1126/science.aaz2449 PMC7380553 32327568

[16] Matsumoto M Minami M Takeda K Sakao Y Akira S. Ectopic expression of chop (Gadd153) induces apoptosis in M1 myeloblastic leukemia cells. FEBS Lett (1996) 395(2-3):143–7. doi: 10.1016/0014-5793(96)01016-2 8898082

[17] Oyadomari S Mori M. Roles of Chop/Gadd153 in endoplasmic reticulum stress. Cell Death Differ (2004) 11(4):381–9. doi: 10.1038/sj.cdd.4401373 14685163

[18] Tabas I Ron D. Integrating the mechanisms of apoptosis induced by endoplasmic reticulum stress. Nat Cell Biol (2011) 13(3):184–90. doi: 10.1038/ncb0311-184 PMC3107571 21364565

[19] Khan MM Yang W-L Wang P. Endoplasmic reticulum stress in sepsis. Shock (2015) 44(4):294–304. doi: 10.1097/SHK.0000000000000425 26125088 PMC4575622

[20] Lee J Ozcan U. Unfolded protein response signaling and metabolic diseases. J Biol Chem (2014) 289(3):1203–11. doi: 10.1074/jbc.R113.534743 PMC3894306 24324257

[21] Madden E Logue SE Healy SJ Manie S Samali A. The role of the unfolded protein response in cancer progression: From oncogenesis to chemoresistance. Biol Cell (2019) 111(1):1–17. doi: 10.1111/boc.201800050 30302777

[22] Ma T Han L Gao Y Li L Shang X Hu W. The endoplasmic reticulum stress-mediated apoptosis signal pathway is involved in sepsis-induced abnormal lymphocyte apoptosis. Eur Surg Res (2008) 41(2):219–25. doi: 10.1159/000135631 18509246

[23] Ferlito M Wang Q Fulton WB Colombani PM Marchionni L Fox-Talbot K. Hydrogen sulfide increases survival during sepsis: Protective effect of chop inhibition. J Immunol (2014) 192(4):1806–14. doi: 10.4049/jimmunol.1300835 PMC3946246 24403532

[24] Sun S Duan Z Wang X Chu C Yang C Chen F. Neutrophil extracellular traps impair intestinal barrier functions in sepsis by regulating Tlr9-mediated endoplasmic reticulum stress pathway. Cell Death Dis (2021) 12(6):606. doi: 10.1038/s41419-021-03896-1 34117211 PMC8195983

[25] Kim S Joe Y Park S-U Jeong SO Kim J-K Park SH. Induction of endoplasmic reticulum stress under endotoxin tolerance increases inflammatory responses and decreases pseudomonas aeruginosa pneumonia. J Leukoc Biol (2018) 104(5):1003–12. doi: 10.1002/JLB.3A0317-106RRR 29924419

[26] Diao L Marshall AH Dai X Bogdanovic E Abdullahi A Amini-Nik S. Burn plus lipopolysaccharide augments endoplasmic reticulum stress and Nlrp3 inflammasome activation and reduces pgc-1α in liver. Shock (2014) 41(2):138–44. doi: 10.1097/SHK.0000000000000075 PMC4635464 24434416

[27] Qian WJ Cheng QH. Endoplasmic reticulum stress-mediated apoptosis signal pathway is involved in sepsis-induced liver injury. Int J Clin Exp Pathol (2017) 10(9):9990–7. PMC6965985 31966888

[28] Wang Y Zhou X Zhao D Wang X Gurley EC Liu R. Berberine inhibits free fatty acid and lps-induced inflammation *Via* modulating er stress response in macrophages and hepatocytes. PloS One (2020) 15(5):e0232630-e. doi: 10.1371/journal.pone.0232630 32357187 PMC7194368

[29] Dubois V Gheeraert C Vankrunkelsven W Dubois-Chevalier J Dehondt H Bobowski-Gerard M. Endoplasmic reticulum stress actively suppresses hepatic molecular identity in damaged liver. Mol Syst Biol (2020) 16(5):e9156. doi: 10.15252/msb.20199156 32407006 PMC7224309

[30] Kim HJ Jeong JS Kim SR Park SY Chae HJ Lee YC. Inhibition of endoplasmic reticulum stress alleviates lipopolysaccharide-induced lung inflammation through modulation of Nf-Kb/Hif-1α signaling pathway. Sci Rep (2013) 3:1142. doi: 10.1038/srep01142 23359618 PMC3556596

[31] Chen X Wang Y Xie X Chen H Zhu Q Ge Z. Heme oxygenase-1 reduces sepsis-induced endoplasmic reticulum stress and acute lung injury. Mediators Inflammation (2018) 2018:9413876. doi: 10.1155/2018/9413876 PMC6022325 30013453

[32] Ferrè S Deng Y Huen SC Lu CY Scherer PE Igarashi P. Renal tubular cell spliced X-box binding protein 1 (Xbp1s) has a unique role in sepsis-induced acute kidney injury and inflammation. Kidney Int (2019) 96(6):1359–73. doi: 10.1016/j.kint.2019.06.023 PMC7286357 31601454

[33] Hato T Maier B Syed F Myslinski J Zollman A Plotkin Z. Bacterial sepsis triggers an antiviral response that causes translation shutdown. J Clin Invest (2019) 129(1):296–309. doi: 10.1172/JCI123284 30507610 PMC6307966

[34] Li L Peng X Guo L Zhao Y Cheng Q. Sepsis causes heart injury through endoplasmic reticulum stress-mediated apoptosis signaling pathway. Int J Clin Exp Pathol (2020) 13(5):964–71. PMC7270664 32509067

[35] Wang Y Jasper H Toan S Muid D Chang X Zhou H. Mitophagy coordinates the mitochondrial unfolded protein response to attenuate inflammation-mediated myocardial injury. Redox Biol (2021) 45:102049. doi: 10.1016/j.redox.2021.102049 34174558 PMC8246635

[36] Vary TC Deiter G Lang CH. Cytokine-triggered decreases in levels of phosphorylated eukaryotic initiation factor 4g in skeletal muscle during sepsis. Shock (2006) 26(6):631–6. doi: 10.1097/01.shk.0000230299.78515.2c 17117141

[37] Orellana RA Wilson FA Gazzaneo MC Suryawan A Davis TA Nguyen HV. Sepsis and development impede muscle protein synthesis in neonatal pigs by different ribosomal mechanisms. Pediatr Res (2011) 69(6):473–8. doi: 10.1203/PDR.0b013e3182176da1 PMC3090498 21364490

[38] Ron D Walter P. Signal integration in the endoplasmic reticulum unfolded protein response. Nat Rev Mol Cell Biol (2007) 8(7):519–29. doi: 10.1038/nrm2199 17565364

[39] Harding HP Zhang Y Ron D. Protein translation and folding are coupled by an endoplasmic-Reticulum-Resident kinase. Nature (1999) 397(6716):271–4. doi: 10.1038/16729 9930704

[40] Ma Q. Role of Nrf2 in oxidative stress and toxicity. Annu Rev Pharmacol Toxicol (2013) 53:401–26. doi: 10.1146/annurev-pharmtox-011112-140320 PMC4680839 23294312

[41] Harding HP Zhang Y Zeng H Novoa I Lu PD Calfon M. An integrated stress response regulates amino acid metabolism and resistance to oxidative stress. Mol Cell (2003) 11(3):619–33. doi: 10.1016/s1097-2765(03)00105-9 12667446

[42] Prostko CR Dholakia JN Brostrom MA Brostrom CO. Activation of the double-stranded rna-regulated protein kinase by depletion of endoplasmic reticular calcium stores. J Biol Chem (1995) 270(11):6211–5. doi: 10.1074/jbc.270.11.6211 7890757

[43] Dever TE Feng L Wek RC Cigan AM Donahue TF Hinnebusch AG. Phosphorylation of initiation factor 2 alpha by protein kinase Gcn2 mediates gene-specific translational control of Gcn4 in yeast. Cell (1992) 68(3):585–96. doi: 10.1016/0092-8674(92)90193-g 1739968

[44] Ranu RS. Regulation of protein synthesis in rabbit reticulocyte lysates: The heme-regulated protein kinase (Hri) and double stranded rna induced protein kinase (Dri) phosphorylate the same Site(S) on initiation factor eif-2. Biochem Biophys Res Commun (1979) 91(4):1437–44. doi: 10.1016/0006-291X(79)91227-0 526314

[45] Zhang P McGrath BC Reinert J Olsen DS Lei L Gill S. The Gcn2 Eif2alpha kinase is required for adaptation to amino acid deprivation in mice. Mol Cell Biol (2002) 22(19):6681–8. doi: 10.1128/mcb.22.19.6681-6688.2002 PMC134046 12215525

[46] Abdel-Nour M Carneiro LAM Downey J Tsalikis J Outlioua A Prescott D. The heme-regulated inhibitor is a cytosolic sensor of protein misfolding that controls innate immune signaling. Science (2019) 365(6448):eaaw4144. doi: 10.1126/science.aaw4144 31273097 PMC10433729

[47] Pakos-Zebrucka K Koryga I Mnich K Ljujic M Samali A Gorman AM. The integrated stress response. EMBO Rep (2016) 17(10):1374–95. doi: 10.15252/embr.201642195 PMC5048378 27629041

[48] Costa-Mattioli M Walter P. The integrated stress response: From mechanism to disease. Science (2020) 368(6489):1–11. doi: 10.1126/science.aat5314 PMC8997189 32327570

[49] Harding HP Zhang Y Bertolotti A Zeng H Ron D. Perk is essential for translational regulation and cell survival during the unfolded protein response. Mol Cell (2000) 5(5):897–904. doi: 10.1016/S1097-2765(00)80330-5 10882126

[50] Scheuner D Song B McEwen E Liu C Laybutt R Gillespie P. Translational control is required for the unfolded protein response and in vivo glucose homeostasis. Mol Cell (2001) 7(6):1165–76. doi: 10.1016/s1097-2765(01)00265-9 11430820

[51] Rzymski T Milani M Singleton DC Harris AL. Role of Atf4 in regulation of autophagy and resistance to drugs and hypoxia. Cell Cycle (2009) 8(23):3838–47. doi: 10.4161/cc.8.23.10086 19887912

[52] Akai R Hosoda A Yoshino M Iwawaki T. Constitutive role of Gadd34 and crep in cancellation of phospho-Eif2α-Dependent translational attenuation and insulin biosynthesis in pancreatic β cells. Genes Cells (2015) 20(11):871–86. doi: 10.1111/gtc.12279 26314560

[53] Lee YY Cevallos RC Jan E. An upstream open reading frame regulates translation of Gadd34 during cellular stresses that induce Eif2alpha phosphorylation. J Biol Chem (2009) 284(11):6661–73. doi: 10.1074/jbc.M806735200 PMC2652341 19131336

[54] Novoa I Zeng H Harding HP Ron D. Feedback inhibition of the unfolded protein response by Gadd34-mediated dephosphorylation of Eif2alpha. J Cell Biol (2001) 153(5):1011–22. doi: 10.1083/jcb.153.5.1011 PMC2174339 11381086

[55] Hu H Tian M Ding C Yu S. The C/Ebp homologous protein (Chop) transcription factor functions in endoplasmic reticulum stress-induced apoptosis and microbial infection. Front Immunol (2019) 9:3083(3083). doi: 10.3389/fimmu.2018.03083 30662442 PMC6328441

[56] Marciniak SJ Yun CY Oyadomari S Novoa I Zhang Y Jungreis R. Chop induces death by promoting protein synthesis and oxidation in the stressed endoplasmic reticulum. Genes Dev (2004) 18(24):3066–77. doi: 10.1101/gad.1250704 PMC535917 15601821

[57] Puthalakath H O'Reilly LA Gunn P Lee L Kelly PN Huntington ND. Er stress triggers apoptosis by activating Bh3-only protein bim. Cell (2007) 129(7):1337–49. doi: 10.1016/j.cell.2007.04.027 17604722

[58] Zou W Yue P Khuri FR Sun S-Y. Coupling of endoplasmic reticulum stress to cddo-Me-Induced up-regulation of death receptor 5 *Via* a chop-dependent mechanism involving jnk activation. Cancer Res (2008) 68(18):7484–92. doi: 10.1158/0008-5472.CAN-08-1318 PMC2597446 18794136

[59] Bartoszewska S Collawn JF. Unfolded protein response (Upr) integrated signaling networks determine cell fate during hypoxia. Cell Mol Biol Lett (2020) 25(1):18. doi: 10.1186/s11658-020-00212-1 32190062 PMC7071609

[60] Madhusudhan T Wang H Dong W Ghosh S Bock F Thangapandi VR. Defective podocyte insulin signalling through P85-Xbp1 promotes Atf6-dependent maladaptive er-stress response in diabetic nephropathy. Nat Commun (2015) 6(1):6496. doi: 10.1038/ncomms7496 25754093 PMC4366504

[61] Yoshida H Okada T Haze K Yanagi H Yura T Negishi M. Atf6 activated by proteolysis binds in the presence of nf-y (Cbf) directly to the cis-acting element responsible for the mammalian unfolded protein response. Mol Cell Biol (2000) 20(18):6755–67. doi: 10.1128/mcb.20.18.6755-6767.2000 PMC86199 10958673

[62] Guo FJ Xiong Z Lu X Ye M Han X Jiang R. Atf6 upregulates Xbp1s and inhibits er stress-mediated apoptosis in osteoarthritis cartilage. Cell Signal (2014) 26(2):332–42. doi: 10.1016/j.cellsig.2013.11.018 24269637

[63] Yang H Niemeijer M van de Water B Beltman JB. Atf6 is a critical determinant of chop dynamics during the unfolded protein response. iScience (2020) 23(2):100860. doi: 10.1016/j.isci.2020.100860 32058971 PMC7005498

[64] Li Y Guo Y Tang J Jiang J Chen Z. New insights into the roles of chop-induced apoptosis in er stress. Acta Biochim Biophys Sin (2014) 46(8):629–40. doi: 10.1093/abbs/gmu048 25016584

[65] Cullinan SB Diehl JA. Perk-dependent activation of Nrf2 contributes to redox homeostasis and cell survival following endoplasmic reticulum stress *. J Biol Chem (2004) 279(19):20108–17. doi: 10.1074/jbc.M314219200 14978030

[66] Cullinan SB Zhang D Hannink M Arvisais E Kaufman RJ Diehl JA. Nrf2 is a direct perk substrate and effector of perk-dependent cell survival. Mol Cell Biol (2003) 23(20):7198–209. doi: 10.1128/mcb.23.20.7198-7209.2003 PMC230321 14517290

[67] Sarcinelli C Dragic H Piecyk M Barbet V Duret C Barthelaix A. Atf4-dependent Nrf2 transcriptional regulation promotes antioxidant protection during endoplasmic reticulum stress. Cancers (2020) 12(3):569. doi: 10.3390/cancers12030569 PMC7139862 32121537

[68] Ye J Rawson RB Komuro R Chen X Davé UP Prywes R. Er stress induces cleavage of membrane-bound Atf6 by the same proteases that process srebps. Mol Cell (2000) 6(6):1355–64. doi: 10.1016/s1097-2765(00)00133-7 11163209

[69] Haze K Yoshida H Yanagi H Yura T Mori K. Mammalian transcription factor Atf6 is synthesized as a transmembrane protein and activated by proteolysis in response to endoplasmic reticulum stress. Mol Biol Cell (1999) 10(11):3787–99. doi: 10.1091/mbc.10.11.3787 PMC25679 10564271

[70] Bertolotti A Zhang Y Hendershot LM Harding HP Ron D. Dynamic interaction of bip and er stress transducers in the unfolded-protein response. Nat Cell Biol (2000) 2(6):326–32. doi: 10.1038/35014014 10854322

[71] Vitale M Bakunts A Orsi A Lari F Tadè L Danieli A. Inadequate bip availability defines endoplasmic reticulum stress. eLife (2019) 8:e41168. doi: 10.7554/eLife.41168 30869076 PMC6417858

[72] Kopp MC Larburu N Durairaj V Adams CJ Ali MMU. Upr proteins Ire1 and perk switch bip from chaperone to er stress sensor. Nat Struct Mol Biol (2019) 26(11):1053–62. doi: 10.1038/s41594-019-0324-9 PMC6858872 31695187

[73] Lewy TG Grabowski JM Bloom ME. Bip: Master regulator of the unfolded protein response and crucial factor in flavivirus biology. Yale J Biol Med (2017) 90(2):291–300. 28656015 PMC5482305

[74] Carrara M Prischi F Nowak PR Kopp MC Ali MM. Noncanonical binding of bip atpase domain to Ire1 and perk is dissociated by unfolded protein Ch1 to initiate er stress signaling. Elife (2015) 4:1–16. doi: 10.7554/eLife.03522 PMC4337721 25692299

[75] Maurel M Chevet E Tavernier J Gerlo S. Getting ridd of rna: Ire1 in cell fate regulation. Trends Biochem Sci (2014) 39(5):245–54. doi: 10.1016/j.tibs.2014.02.008 24657016

[76] Almanza A Mnich K Blomme A Robinson CM Rodriguez-Blanco G Kierszniowska S. Regulated Ire1α-dependent decay (Ridd)-mediated reprograming of lipid metabolism in cancer. Nat Commun (2022) 13(1):2493. doi: 10.1038/s41467-022-30159-0 35524156 PMC9076827

[77] Duwaerts CC Siao K Soon RK Jr. Her C Iwawaki T Kohno K. Hepatocyte-specific deletion of Xbp1 sensitizes mice to liver injury through hyperactivation of Ire1α. Cell Death Differ (2021) 28(5):1455–65. doi: 10.1038/s41418-020-00671-1 PMC8166833 33219328

[78] Ishikawa T Kashima M Nagano AJ Ishikawa-Fujiwara T Kamei Y Todo T. Unfolded protein response transducer Ire1-mediated signaling independent of Xbp1 mrna splicing is not required for growth and development of medaka fish. eLife (2017) 6:e26845. doi: 10.7554/eLife.26845 28952924 PMC5636610

[79] Zhou Y Lee J Reno CM Sun C Park SW Chung J. Regulation of glucose homeostasis through a Xbp-1–Foxo1 interaction. Nat Med (2011) 17(3):356–65. doi: 10.1038/nm.2293 PMC3897616 21317886

[80] Ozcan U Cao Q Yilmaz E Lee AH Iwakoshi NN Ozdelen E. Endoplasmic reticulum stress links obesity, insulin action, and type 2 diabetes. Science (2004) 306(5695):457–61. doi: 10.1126/science.1103160 15486293

[81] Lee AH Scapa EF Cohen DE Glimcher LH. Regulation of hepatic lipogenesis by the transcription factor Xbp1. Science (2008) 320(5882):1492–6. doi: 10.1126/science.1158042 PMC3620093 18556558

[82] Hollien J Weissman JS. Decay of endoplasmic reticulum-localized mrnas during the unfolded protein response. Science (2006) 313(5783):104–7. doi: 10.1126/science.1129631 16825573

[83] Upton J-P Austgen K Nishino M Coakley KM Hagen A Han D. Caspase-2 cleavage of bid is a critical apoptotic signal downstream of endoplasmic reticulum stress. Mol Cell Biol (2008) 28(12):3943–51. doi: 10.1128/MCB.00013-08 PMC2423129 18426910

[84] Hollien J Lin JH Li H Stevens N Walter P Weissman JS. Regulated Ire1-dependent decay of messenger rnas in mammalian cells. J Cell Biol (2009) 186(3):323–31. doi: 10.1083/jcb.200903014 PMC2728407 19651891

[85] Lerner Alana G Upton J-P Praveen PVK Ghosh R Nakagawa Y Igbaria A. IRE1α induces thioredoxin-interacting protein to activate the Nlrp3 inflammasome and promote programmed cell death under irremediable er stress. Cell Metab (2012) 16(2):250–64. doi: 10.1016/j.cmet.2012.07.007 PMC4014071 22883233

[86] Park SM Kang TI So JS. Roles of Xbp1s in transcriptional regulation of target genes. Biomedicines (2021) 9(7):1–26. doi: 10.3390/biomedicines9070791 PMC8301375 34356855

[87] So J-S Hur Kyu Y Tarrio M Ruda V Frank-Kamenetsky M Fitzgerald K. Silencing of lipid metabolism genes through Ire1&-mediated mrna decay lowers plasma lipids in mice. Cell Metab (2012) 16(4):487–99. doi: 10.1016/j.cmet.2012.09.004 PMC3475419 23040070

[88] Upton JP Wang L Han D Wang ES Huskey NE Lim L. Ire1α cleaves select micrornas during er stress to derepress translation of proapoptotic caspase-2. Science (2012) 338(6108):818–22. doi: 10.1126/science.1226191 PMC3742121 23042294

[89] Zeng T Peng L Chao H Xi H Fu B Wang Y. Ire1α-Traf2-Ask1 complex-mediated endoplasmic reticulum stress and mitochondrial dysfunction contribute to Cxc195-induced apoptosis in human bladder carcinoma T24 cells. Biochem Biophys Res Commun (2015) 460(3):530–6. doi: 10.1016/j.bbrc.2015.03.064 25797626

[90] Medzhitov R. The spectrum of inflammatory responses. Science (2021) 374(6571):1070–5. doi: 10.1126/science.abi5200 34822279

[91] Jentho E Weis S. Damps and innate immune training. Front Immunol (2021) 12:699563. doi: 10.3389/fimmu.2021.699563 34745089 PMC8569823

[92] Netea MG Balkwill F Chonchol M Cominelli F Donath MY Giamarellos-Bourboulis EJ. A guiding map for inflammation. Nat Immunol (2017) 18(8):826–31. doi: 10.1038/ni.3790 PMC5939996 28722720

[93] Akira S Uematsu S Takeuchi O. Pathogen recognition and innate immunity. Cell (2006) 124(4):783–801. doi: 10.1016/j.cell.2006.02.015 16497588

[94] Smith JA. Regulation of cytokine production by the unfolded protein response; implications for infection and autoimmunity. Front Immunol (2018) 9:422. doi: 10.3389/fimmu.2018.00422 29556237 PMC5844972

[95] Martinon F Chen X Lee A-H Glimcher LH. Tlr activation of the transcription factor Xbp1 regulates innate immune responses in macrophages. Nat Immunol (2010) 11(5):411–8. doi: 10.1038/ni.1857 PMC3113706 20351694

[96] Huang C Hedl M Ranjan K Abraham C. Lacc1 required for Nod2-induced, er stress-mediated innate immune outcomes in human macrophages and Lacc1 risk variants modulate these outcomes. Cell Rep (2019) 29(13):4525–39.e4. doi: 10.1016/j.celrep.2019.11.105 31875558 PMC7372507

[97] Keestra-Gounder AM Byndloss MX Seyffert N Young BM Chávez-Arroyo A Tsai AY. Nod1 and Nod2 signalling links er stress with inflammation. Nature (2016) 532(7599):394–7. doi: 10.1038/nature17631 PMC4869892 27007849

[98] Jong M Starr T Winter MG Hartigh A Child R Knodler LA. Sensing of bacterial type iv secretion *Via* the unfolded protein response. mBio (2013) 4(1):e00418-12. doi: 10.1128/mBio.00418-12 23422410 PMC3624511

[99] Yahiro K Ogura K Tsutsuki H Iyoda S Ohnishi M Moss JA. Novel endoplasmic stress mediator, kelch domain containing 7b (Klhdc7b), increased harakiri (Hrk) in the subab-induced apoptosis signaling pathway. Cell Death Discovery (2021) 7(1):360. doi: 10.1038/s41420-021-00753-0 34799565 PMC8605022

[100] Yamazaki H Hiramatsu N Hayakawa K Tagawa Y Okamura M Ogata R. Activation of the akt-Nf-Kappab pathway by subtilase cytotoxin through the Atf6 branch of the unfolded protein response. J Immunol (2009) 183(2):1480–7. doi: 10.4049/jimmunol.0900017 PMC2762936 19561103

[101] Bronner Denise N Abuaita Basel H Chen X Fitzgerald Katherine A Nuñez G He Y. Endoplasmic reticulum stress activates the inflammasome *Via* Nlrp3- and caspase-2-Driven mitochondrial damage. Immunity (2015) 43(3):451–62. doi: 10.1016/j.immuni.2015.08.008 PMC4582788 26341399

[102] Osorio F Tavernier SJ Hoffmann E Saeys Y Martens L Vetters J. The unfolded-Protein-Response sensor ire-1α regulates the function of Cd8α+ dendritic cells. Nat Immunol (2014) 15(3):248–57. doi: 10.1038/ni.2808 24441789

[103] Iwakoshi NN Pypaert M Glimcher LH. The transcription factor xbp-1 is essential for the development and survival of dendritic cells. J Exp Med (2007) 204(10):2267–75. doi: 10.1084/jem.20070525 PMC2118458 17875675

[104] Tavernier SJ Osorio F Vandersarren L Vetters J Vanlangenakker N Van Isterdael G. Regulated Ire1-dependent mrna decay sets the threshold for dendritic cell survival. Nat Cell Biol (2017) 19(6):698–710. doi: 10.1038/ncb3518 28459443 PMC5563826

[105] Gass JN Gunn KE Sriburi R Brewer JW. Stressed-out b cells? plasma-cell differentiation and the unfolded protein response. Trends Immunol (2004) 25(1):17–24. doi: 10.1016/j.it.2003.11.004 14698280

[106] Gass JN Jiang HY Wek RC Brewer JW. The unfolded protein response of b-lymphocytes: Perk-independent development of antibody-secreting cells. Mol Immunol (2008) 45(4):1035–43. doi: 10.1016/j.molimm.2007.07.029 PMC2677759 17822768

[107] Reimold AM Iwakoshi NN Manis J Vallabhajosyula P Szomolanyi-Tsuda E Gravallese EM. Plasma cell differentiation requires the transcription factor xbp-1. Nature (2001) 412(6844):300–7. doi: 10.1038/35085509 11460154

[108] Iwakoshi NN Lee AH Vallabhajosyula P Otipoby KL Rajewsky K Glimcher LH. Plasma cell differentiation and the unfolded protein response intersect at the transcription factor xbp-1. Nat Immunol (2003) 4(4):321–9. doi: 10.1038/ni907 12612580

[109] Gass JN Gifford NM Brewer JW. Activation of an unfolded protein response during differentiation of antibody-secreting b cells *. J Biol Chem (2002) 277(50):49047–54. doi: 10.1074/jbc.M205011200 12374812

[110] Clavier T Grangé S Pressat-Laffouilhere T Besnier E Renet S Fraineau S. Gene expression of protein tyrosine phosphatase 1b and endoplasmic reticulum stress during septic shock. Front Med (2019) 6:240(240). doi: 10.3389/fmed.2019.00240 PMC6839276 31737637

[111] van der Poll T Shankar-Hari M Wiersinga WJ. The immunology of sepsis. Immunity (2021) 54(11):2450–64. doi: 10.1016/j.immuni.2021.10.012 34758337

[112] Gustave C-A Gossez M Demaret J Rimmelé T Lepape A Malcus C. Septic shock shapes b cell response toward an exhausted-Like/Immunoregulatory profile in patients. J Immunol (2018) 200(7):2418–25. doi: 10.4049/jimmunol.1700929 29459404

[113] Shankar-Hari M Fear D Lavender P Mare T Beale R Swanson C. Activation-associated accelerated apoptosis of memory b cells in critically ill patients with sepsis. Crit Care Med (2017) 45(5):875–82. doi: 10.1097/ccm.0000000000002380 28296810

[114] Huen SC. Metabolism as disease tolerance: Implications for sepsis-associated acute kidney injury. Nephron (2021) 146(3):291–294. doi: 10.1159/000516877 PMC8695642 34161955

[115] Ping F Li Y Cao Y Shang J Zhang Z Yuan Z. Metabolomics analysis of the development of sepsis and potential biomarkers of sepsis-induced acute kidney injury. Oxid Med Cell Longev (2021) 2021:6628847. doi: 10.1155/2021/6628847 33981387 PMC8088350

[116] Strnad P Tacke F Koch A Trautwein C. Liver — guardian, modifier and target of sepsis. Nat Rev Gastroenterol Hepatol (2017) 14(1):55–66. doi: 10.1038/nrgastro.2016.168 27924081

[117] Protzer U Maini MK Knolle PA. Living in the liver: Hepatic infections. Nat Rev Immunol (2012) 12(3):201–13. doi: 10.1038/nri3169 22362353

[118] Canabal JM Kramer DJ. Management of sepsis in patients with liver failure. Curr Opin Crit Care (2008) 14(2):189–97. doi: 10.1097/MCC.0b013e3282f6a435 18388682

[119] Nesseler N Launey Y Aninat C White J Corlu A Pieper K. Liver dysfunction is associated with long-term mortality in septic shock. Am J Respir Crit Care Med (2016) 193(3):335–7. doi: 10.1164/rccm.201508-1660LE 26829424

[120] Lei Y Wan S Liu H Zhou H Chen L Yang Y. Arrb1 suppresses the activation of hepatic macrophages *Via* modulating endoplasmic reticulum stress in lipopolysaccharide-induced acute liver injury. Cell Death Discovery (2021) 7(1):223. doi: 10.1038/s41420-021-00615-9 34455423 PMC8403172

[121] Rao J Yue S Fu Y Zhu J Wang X Busuttil RW. Am J transplant. Am J Transplant (2014) 14(7):1552–61. doi: 10.1111/ajt.12711 PMC4074706 24903305

[122] Michalopoulos GK Bhushan B. Liver regeneration: Biological and pathological mechanisms and implications. Nat Rev Gastroenterol Hepatol (2021) 18(1):40–55. doi: 10.1038/s41575-020-0342-4 32764740

[123] Liu Y Shao M Wu Y Yan C Jiang S Liu J. Role for the endoplasmic reticulum stress sensor Ire1α in liver regenerative responses. J Hepatol (2015) 62(3):590–8. doi: 10.1016/j.jhep.2014.10.022 25457211

[124] Englert JA Bobba C Baron RM. Integrating molecular pathogenesis and clinical translation in sepsis-induced acute respiratory distress syndrome. JCI Insight (2019) 4(2):1–13. doi: 10.1172/jci.insight.124061 PMC6413834 30674720

[125] Zhou X Liao Y. Gut-lung crosstalk in sepsis-induced acute lung injury. Front Microbiol (2021) 12:779620. doi: 10.3389/fmicb.2021.779620 35003009 PMC8733643

[126] Jentho E Ruiz-Moreno C Novakovic B Kourtzelis I Megchelenbrink WL Martins R. Trained innate immunity, long-lasting epigenetic modulation, and skewed myelopoiesis by heme. PNAS (2021) 118(42):e2102698118. doi: 10.1073/pnas.2102698118 34663697 PMC8545490

[127] Lin Q Weis S Yang G Weng YH Helston R Rish K. Heme oxygenase-1 protein localizes to the nucleus and activates transcription factors important in oxidative stress. J Biol Chem (2007) 282(28):20621–33. doi: 10.1074/jbc.M607954200 17430897

[128] Huang P Peslak SA Lan X Khandros E Yano JA Sharma M. The hri-regulated transcription factor Atf4 activates Bcl11a transcription to silence fetal hemoglobin expression. Blood (2020) 135(24):2121–32. doi: 10.1182/blood.2020005301 PMC7290097 32299090

[129] Suragani RN Zachariah RS Velazquez JG Liu S Sun CW Townes TM. Heme-regulated Eif2α kinase activated Atf4 signaling pathway in oxidative stress and erythropoiesis. Blood (2012) 119(22):5276–84. doi: 10.1182/blood-2011-10-388132 PMC3369616 22498744

[130] Reichard JF Motz GT Puga A. Heme oxygenase-1 induction by Nrf2 requires inactivation of the transcriptional repressor Bach1. Nucleic Acids Res (2007) 35(21):7074–86. doi: 10.1093/nar/gkm638 PMC2175339 17942419

[131] Joffre J Hellman J Ince C Ait-Oufella H. Endothelial responses in sepsis. Am J Respir Crit Care Med (2020) 202(3):361–70. doi: 10.1164/rccm.201910-1911TR 32101446

[132] Ince C Mayeux PR Nguyen T Gomez H Kellum JA Ospina-Tascón GA. The endothelium in sepsis. Shock (2016) 45(3):259–70. doi: 10.1097/shk.0000000000000473 PMC5281063 26871664

[133] Lenna S Han R Trojanowska M. Endoplasmic reticulum stress and endothelial dysfunction. IUBMB Life (2014) 66(8):530–7. doi: 10.1002/iub.1292 PMC4181710 25130181

[134] Barabutis N. Unfolded protein response: A regulator of the endothelial barrier. Endocr Metab Sci (2021) 3:1–2. doi: 10.1016/j.endmts.2021.100092 PMC8009497 33796874

[135] Hollenberg SM Singer M. Pathophysiology of sepsis-induced cardiomyopathy. Nat Rev Cardiol (2021) 18(6):424–34. doi: 10.1038/s41569-020-00492-2 33473203

[136] Frencken JF Donker DW Spitoni C Koster-Brouwer ME Soliman IW Ong DSY. Myocardial injury in patients with sepsis and its association with long-term outcome. Circ Cardiovasc Qual Outcomes (2018) 11(2):e004040. doi: 10.1161/CIRCOUTCOMES.117.004040 29378734

[137] Habimana R Choi I Cho HJ Kim D Lee K Jeong I. Sepsis-induced cardiac dysfunction: A review of pathophysiology. Acute Crit Care (2020) 35(2):57–66. doi: 10.4266/acc.2020.00248 32506871 PMC7280799

[138] Zhang B Liu Y Zhang JS Zhang XH Chen WJ Yin XH. Cortistatin protects myocardium from endoplasmic reticulum stress induced apoptosis during sepsis. Mol Cell Endocrinol (2015) 406:40–8. doi: 10.1016/j.mce.2015.02.016 25727193

[139] Owen AM Patel SP Smith JD Balasuriya BK Mori SF Hawk GS. Chronic muscle weakness and mitochondrial dysfunction in the absence of sustained atrophy in a preclinical sepsis model. eLife (2019) 8:e49920. doi: 10.7554/eLife.49920 31793435 PMC6890461

[140] Callahan LA Supinski GS. Sepsis-induced myopathy. Crit Care Med (2009) 37(10 Suppl):S354–S67. doi: 10.1097/CCM.0b013e3181b6e439 PMC3967515 20046121

[141] Lang CH Frost RA Vary TC. Regulation of muscle protein synthesis during sepsis and inflammation. Am J Physiol Endocrinol Metab (2007) 293(2):E453–9. doi: 10.1152/ajpendo.00204.2007 17505052

[142] Metzing UB von Loeffelholz C Steidl R Romeike B Winkler R Rauchfuß F. Endoplasmic reticulum stress and the unfolded protein response in skeletal muscle of subjects suffering from peritoneal sepsis. Sci Rep (2022) 12(1):504. doi: 10.1038/s41598-021-04517-9 35017615 PMC8752775

[143] Vary TC Kimball SR. Sepsis-induced changes in protein synthesis: Differential effects on fast- and slow-twitch muscles. Am J Physiol (1992) 262(6 Pt 1):C1513–9. doi: 10.1152/ajpcell.1992.262.6.C1513 1377447

[144] Amorim IS Lach G Gkogkas CG. The role of the eukaryotic translation initiation factor 4e (Eif4e) in neuropsychiatric disorders. Front Genet (2018) 9:561. doi: 10.3389/fgene.2018.00561 30532767 PMC6265315

[145] Pelletier J Graff J Ruggero D Sonenberg N. Targeting the Eif4f translation initiation complex: A critical nexus for cancer development. Cancer Res (2015) 75(2):250–63. doi: 10.1158/0008-5472.Can-14-2789 PMC4299928 25593033

[146] Kazi AA Pruznak AM Frost RA Lang CH. Sepsis-induced alterations in protein-protein interactions within mtor complex 1 and the modulating effect of leucine on muscle protein synthesis. Shock (2011) 35(2):117–25. doi: 10.1097/shk.0b013e3181ecb57c PMC2995824 20577146

[147] Bohnert KR Gallot YS Sato S Xiong G Hindi SM Kumar A. Inhibition of er stress and unfolding protein response pathways causes skeletal muscle wasting during cancer cachexia. FASEB J (2016) 30(9):3053–68. doi: 10.1096/fj.201600250RR PMC5001510 27206451

